# Successful Ziprasidone Monotherapy in a Case of Delusional Parasitosis: A One-Year Followup

**DOI:** 10.1155/2013/913248

**Published:** 2013-05-16

**Authors:** Domenico De Berardis, Nicola Serroni, Stefano Marini, Gabriella Rapini, Alessandro Valchera, Michele Fornaro, Monica Mazza, Felice Iasevoli, Giovanni Martinotti, Massimo Di Giannantonio

**Affiliations:** ^1^National Health Service, Department of Mental Health, Psychiatric Service of Diagnosis and Treatment, Hospital “G. Mazzini”, ASL 4, 64100 Teramo, Italy; ^2^Department of Neuroscience and Imaging, Chair of Psychiatry, University “G. D'Annunzio”, 66100 Chieti, Italy; ^3^Hermanas Hospitalarias, FoRiPsi, Villa S. Giuseppe Hospital, 63100 Ascoli Piceno, Italy; ^4^Department of “Scienze della Formazione”, University of Catania, 95121 Catania, Italy; ^5^Department of Health Science, University of L'Aquila, 67100 L'Aquila, Italy; ^6^Laboratory of Molecular Psychiatry and Psychopharmacotherapeutics, Section of Psychiatry, Department of Neuroscience, University School of Medicine “Federico II”, 80131 Naples, Italy

## Abstract

Delusional parasitosis is characterized by the false idea that own body is infested by invisible mites, insects, or other parasites. This case report describes a 24-year-old woman with delusional parasitosis who was treated with ziprasidone monotherapy (120 mg/day) with a complete remission of delusion and followed for one year without symptom recurrences. These findings, although preliminary, indicate that further investigation of ziprasidone monotherapy for the treatment of delusional parasitosis is warranted in further trials.

## 1. Introduction

Delusional parasitosis (also known as Ekbom syndrome, delusion of infestation, psychogenic parasitosis, dermatozoenwahn, and dermatozoic delusion) is characterized by the idea that own body is infested by invisible mites, insects, or other parasites [1]. Patients often complain about itching that attribute to the existence of such animals in or under the skin [2]. It is mainly considered a monosymptomatic hypochondriacal psychosis meeting DSM-IV-TR criteria for delusional disorder, somatic type [3], but it may also be associated with other psychiatric or organic diseases [4, 5]. In fact, it should be noted that the diagnosis of primary delusional parasitosis may be suggested only after real parasite infections or other underlying medical or psychiatric comorbid conditions have been ruled out, as delusional parasitosis may be often associated with some physical illnesses, several psychiatric disorders, or substance abuse/intoxications [1, 2, 6]. It has also been reported that delusional parasitosis can also occur as a shared psychotic disorder (folie à deux or folie à trois) and even as by proxy [7]. The patients with untreated primary delusional parasitosis may be at risk of committing suicide [8].

Both typical and atypical antipsychotics have been successfully employed in the treatment of primary delusional parasitosis [9–11] and many sources have recommended the use of the typical antipsychotic pimozide, that seems particularly effective. Contreras-Ferrer et al. [12] described the case of a 73-year-old patient with delusional parasitosis who did not responded completely to pimozide and was successfully treated with ziprasidone augmentation.

However, to date, no information is present on efficacy of ziprasidone monotherapy in the treatment of primary delusional parasitosis. 

## 2. Case Report

The case was a 24-year-old female university student who came to our observation at our outpatient facility in December 2010, after several consultations at the emergency unit of our hospital. She complained that an unknown invisible parasite infestation was causing her generalized chronic pruritus. She reported also pinprick biting sensations several times a day. The onset of symptoms lasted at least from six months. She had consulted several dermatologists and a parasitologist who excluded any ectoparasitosis and/or other dermatological diseases and recommended the girl to consult a psychiatrist. 

At the visit, she expressed a sensation of bugs moving under her skin all over her body. This tactile hallucination was “…initially intermittent but now is almost continuous and are more present when I attend the lessons at university or I'm in the bed…” The patient reported that “…now I avoid to go to university and study…I have destroyed and changed many times all my clothes, bath and bed sheets without results…”. She also complained of seeing the larvae of bugs in her stools but this was not confirmed either by several macroscopic or microscopic examinations or by stool cultures. The patient reported a severe social withdrawal avoiding contact with other people to exclude the risk of infesting them. 

On the day of the visit, the patient was in good physical health, apart from several scratching lesions localized mostly on her arms, forehead, neck and legs. Laboratory test results, including serum electrolytes, chemistry profile, liver and renal function, thyroid function tests, prolactin, serum B12, folate levels, HIV and hepatitis screening, homocysteine, electroencephalogram, electrocardiogram (QT/QTc within normal limits), and chest radiograph, were obtained and were within normal limits. A computed tomography of the brain showed no essential abnormalities and neurological examination was negative. Also substance or alcohol use was ruled out as the personal history and laboratory screening were negative.

We established the diagnosis of primary delusional parasitosis. This patient was administered ziprasidone, titrated to 60 mg twice a day, over the course of a week. The choice of ziprasidone was made considering the request of the patient to prefer a drug with a low propensity to cause weight gain and the potential efficacy of ziprasidone on delusions due to its D2 blockade properties. After one month of ziprasidone monotherapy, the delusion was alleviated with a significant reduction of worry and subjective pruritus. No adverse effects related to ziprasidone were observed. At the end of the second month a full remission of symptomatology was observed with a complete recovery. She was followed bimonthly in our outpatient facility and maintained complete recovery during time. After four months of therapy, she started again to attend lessons at university without problems and, after five months, started working as an office clerk. 

The last observation was made in December 2011: the patient was taking ziprasidone 120 mg/day with a good compliance and absence of parasitosis delusion and without adverse effects including weight gain, metabolic syndrome, hyperprolactinemia, and extrapyramidal symptoms. ECG showed a normal corrected QTc interval. She regularly attends lessons and work.

The patient provided informed consent to present this report.

## 3. Discussion

To date, this was the first described case that showed the efficacy of ziprasidone monotherapy in the treatment of delusional parasitosis. In this case report, we observed a false fixed belief of being infested by parasites characteristic of Ekbom syndrome in a young female patient. We classified the disorder as a primary delusional parasitosis, while secondary delusional parasitosis was excluded on the basis of the anamnesis, laboratory, and instrumental data. Moreover, as delusional parasitosis is often accompanied with a refusal to seek psychiatric care, we decided to use ziprasidone mainly due to a patient' direct request to have a drug with lower propensity to cause weight gain, in order to promote the treatment compliance [5]. 

It has been reported that atypical antipsychotic agents may be effective in the treatment of delusional parasitosis [13], but, to date, this is the first report on efficacy of ziprasidone monotherapy. Ziprasidone is a novel anti-psychotic with a unique pattern of receptor affinity. Ziprasidone is a potent antagonist at the serotonin 5-HT2A, 5-HT1D, 5-HT2C receptors, and dopamine D2 receptor with a high 5-HT2A/D2 ratio, and a potent agonist at 5-HT1A receptors [14]. In this case report, the authors prescribed ziprasidone monotherapy and obtained a dramatic improvement of delusion without developing parkinsonism and weight gain. 

Even if the pathophysiology of delusional parasitosis remains unknown, it has been hypothesized that a decreased striatal dopamine transporter functioning leading to increased extracellular dopamine levels may be the neurobiological background [15, 16]. Moreover, it has been reported that delusional parasitosis may arise during treatment with dopamine agonists or with substances influencing the dopamine transporter [17]. Therefore, delusional parasitosis usually develops due to hyperactivity of dopamine systems especially in the striatum and, predominantly, in the putamen [18]. In our case report, the stabilizing effect of ziprasidone on the dopamine systems may explain why ziprasidone was effective on delusion. Moreover, ziprasidone was well tolerated and no parkinsonism, weight gain, and cardiac side effects were observed [19].

## 4. Conclusions

In conclusion, ziprasidone monotherapy might be effective at least for some patients with delusional parasitosis. It appears to be an adequate alternative to other atypical antipsychotics such as olanzapine and quetiapine that may be associated with the increased risk of developing weight gain and metabolic syndrome that may be of particular concern especially in young females. However, this was merely a single case report and prospective double-blind, placebo-controlled studies are undoubtedly needed.

## References

[1] Hinkle NC (2011). Ekbom syndrome: a delusional condition of ‘bugs in the skin’. *Current Psychiatry Reports*.

[2] Hinkle NC (2010). Ekbom syndrome: the challenge of “invisible bug” infestations. *Annual Review of Entomology*.

[3] Nicolato R, Corrêa H, Romano-Silva MA, Teixeira AL (2006). Delusional parasitosis or Ekbom syndrome: a case series. *General Hospital Psychiatry*.

[4] Bhatia MS, Jagawat T, Choudhary S (2000). Delusional parasitosis: a clinical profile. *International Journal of Psychiatry in Medicine*.

[5] Trabert W (1995). 100 years of delusional parasitosis: meta-analysis of 1,223 case reports. *Psychopathology*.

[6] Martinotti G, Di Iorio G, Sepede G, De Berardis D, De Risio L, Di Giannantonio M (2012). Cannabis use and psychosis: theme introduction. *Current Pharmaceutical Design*.

[7] Bury JE, Bostwick JM (2010). Iatrogenic delusional parasitosis: a case of physician-patient folie a deux. *General Hospital Psychiatry*.

[8] Marasco V, De Berardis D, Serroni N (2011). Alexithymia and suicide risk among patients with schizophrenia: preliminary findings of a cross-sectional study. *Rivista di Psichiatria*.

[9] Lepping P, Russell I, Freudenmann RW (2007). Antipsychotic treatment of primary delusional parasitosis: systematic review. *British Journal of Psychiatry*.

[10] Freudenmann RW, Lepping P (2008). Second-generation antipsychotics in primary and secondary delusional parasitosis: outcome and efficacy. *Journal of Clinical Psychopharmacology*.

[11] Huber M, Lepping P, Pycha R, Karner M, Schwitzer J, Freudenmann RW (2011). Delusional infestation: treatment outcome with antipsychotics in 17 consecutive patients (using standardized reporting criteria). *General Hospital Psychiatry*.

[12] Contreras-Ferrer P, de Paz NM, Cejas-Mendez MR, Rodríguez-Martín M, Souto R, Bustínduy MG (2012). Ziprasidone in the treatment of delusional parasitosis. *Case Reports in Dermatology*.

[13] Milia A, Mascia MG, Pilia G (2008). Efficacy and safety of quetiapine treatment for delusional parasitosis: experience in an elderly patient. *Clinical Neuropharmacology*.

[14] Stip E, Zhornitsky S, Moteshafi H (2011). Ziprasidone for psychotic disorders: a meta-analysis and systematic review of the relationship between pharmacokinetics, pharmacodynamics, and clinical profile. *Clinical Therapeuthics*.

[15] Huber M, Kirchler E, Karner M, Pycha R (2007). Delusional parasitosis and the dopamine transporter. A new insight of etiology?. *Medical Hypotheses*.

[16] Treuer T, Martenyi F, Karagianis J (2007). Parasitosis, dopaminergic modulation and metabolic disturbances in schizophrenia: evolution of a hypothesis. *Neuroendocrinology Letters*.

[17] Flann S, Shotbolt J, Kessel B (2010). Three cases of delusional parasitosis caused by dopamine agonists. *Clinical and Experimental Dermatology*.

[18] Huber M, Karner M, Kirchler E, Lepping P, Freudenmann RW (2008). Striatal lesions in delusional parasitosis revealed by magnetic resonance imaging. *Progress in Neuro-Psychopharmacology and Biological Psychiatry*.

[19] Bersani G, Gherardelli S, Clemente R (2005). Neurologic soft signs in schizophrenic patients treated with conventional and atypical antipsychotics. *Journal of Clinical Psychopharmacology*.

